# Laughter as a Medicine

**Published:** 1885-03

**Authors:** 


### LAUGHTER AS A MEDICINE.

A short time since, two individuals were lying in a room very sick, one with brain fever and the other with an aggravated case of the mumps. They were so low that watches were needed every night, and it was thought doubtful if either one could recover. A gentleman was engaged to watch over night, his duty being to wake the nurse whenever it became necessary to administer medicine. In the course of the night both watcher and nurse fell asleep. The man with the mumps lay watching the clock, and saw that it was time to give the fever patient his potion. He was unable to speak aloud or to move any portion of his body except his arms, but seizing a pillow, he manage to strike the watcher in the face wlth it. Thus suddenly awakened, the watcher sprang from his seat, falling to the floor, and awoke both the nurse and the fever patient. The incident struck the sick men as very ludicrous, and they laughed heartily at it for some fifteen or twenty minutes. When the doctor came in the morning he found his patients vastly improved; said he never knew so sudden a turn for the better, and now both are up and well. Who says laughter is not the best of medicines. And this reminds the writer of another case. A gentleman was suffering from an ulceration of the throat, which at length became so swollen that his life was despaired of. His household came to his bedside to bid him farewell. Each individual shook hands with the dying man and then went away weeping. Last of all came a pet ape, and shaking the man's hand went away also with its hands over its eyes. It was so ludricrous a sight that the patient was forced to laugh so heartily that the ulcer broke and his life was saved.—*Sanitarian.*

